# Mental health content in the physiotherapy undergraduate curriculum in South Africa

**DOI:** 10.4102/sajp.v80i1.2061

**Published:** 2024-07-31

**Authors:** Marilyn Hooblaul, Oladapo M. Olagbegi, Thayananthee Nadasan

**Affiliations:** 1 Discipline of Physiotherapy, College of Health Sciences, School of Health Sciences, University of KwaZulu-Natal, Durban, South Africa

**Keywords:** mental health, physiotherapy, curriculum, undergraduate programme, South Africa

## Abstract

**Background:**

Knowledge about mental health in physiotherapy practice is essential as mental health can impact physical health. Little is known about the mental health content in the South African physiotherapy undergraduate programme.

**Objectives:**

Our study explored the mental health content in the undergraduate physiotherapy programme at eight universities and the perceptions of academic staff at an identified training institution in South Africa and stakeholders about the relevance of mental health in the undergraduate physiotherapy programme

**Method:**

Our study employed a concurrent-mixed method design, which consisted of: (1) the administration of a survey to academic leaders or lecturers undertaking mental health teaching at universities and (2) an online interview that included stakeholders and a focus group to gauge perceptions of academic staff at an identified institution in South Africa.

**Results:**

Seven of eight universities participated in our study. All the universities had a psychology module and agreed that it is crucial to have mental health content in the curriculum. There is diversity in the mental health content between the universities.

**Conclusion:**

There is a need for consistency in the mental health content at all universities to ensure that all students receive the same skill set to have an impact on the quality of care.

**Clinical implications:**

There is a need to include mental health content at universities offering theoretical and practical undergraduate programmes.

## Introduction

Physiotherapists rehabilitate a wide range of patients in various settings. Therefore, the training in knowledge and skills must cover a broad range of teaching areas (Heaney et al. 2012). Physiotherapy is primarily concerned with a patient’s physical condition (Probst & Skjaerven 2017); however, consideration of the patient’s mental health plays a significant role in treatment (Heaney et al. 2012). Physiotherapy in mental health is based on a 50-year history of evidence-based literature (Kille 2021). In 2006, a group of physiotherapists working in the mental health field formed the ‘International Organisation of Physical Therapy in Mental Health’ IOPTMH (Probst et al. 2019) as a subgroup of the World Confederation of Physical Therapy (WCPT) in 2011, representing 59 nations including South Africa (Stathopoulos 2020). In 2020, the IOPTMH developed an educational standards guideline for mental health curriculum in physiotherapy training programmes (Probst et al. 2020).

Physiotherapy in mental health is often regarded as insignificant in the management of people living with mental illness (PLWMI) and the role and value are unclear among multidisciplinary team members (Kille 2021). One in four people experience a mental health disorder in their lifetime (Pope 2009). The inability to recognise mental health disorders can lead to a delay in physical recovery (Everett, Donaghy & Feaver 2003). It can, therefore, be said that it is vital for physiotherapists to have basic knowledge about mental health. Physiotherapy interventions complement medication and psychotherapy (Kaur & Garnawat 2009). Quality of life can be greatly improved for people with serious mental illness with the intervention of physical activity (Kaur, Masuan & Bhatia 2013). Aerobic exercises have been proven to decrease anxiety and depression (Kaur & Garnawat 2009). Physical activity improves both physical and mental health (Kaur & Garnawat 2009). The physiotherapist’s role in the psychiatric multidisciplinary team is to act as a bridge between physical and mental health, and they are integral in providing treatment for the physical health of PLWMI (Stubbs et al. 2014).

Physiotherapists’ key role is educating patients on the bio-psychosocial benefits of physical activity (Soundy et al. 2014). The physiotherapy goals are based on the SMART principle (specific, measurable, acceptable or attainable, realistic or relevant and time-bound) (Probst & Skjaerven 2017). All goals and treatment strategies are based on the bio-psychosocial model of the International Classification of Functioning (ICF) and the Disability and Health Model (Probst et al. 2019). The model is the framework for describing and organising information on health and disability (World Health Organisation [WHO] 2001). Physiotherapy in mental health emphasises activity and participation and considers the impact of the environment and personal factors that affect treatment.

The legislation and policy commitment are in place to protect the human rights of PLWMI (RSA 2002). *The Mental Health Care Act of 17* (2002) (MHCA) was promulgated in 2004; this policy was formulated to break the apartheid practices that existed in the health system prior to 1994 that discriminated against PLWMI (Ramlall 2012). The policy aims to integrate mental health into primary healthcare (PHC) (Meyer et al. 2019) and to ensure that mental healthcare users (MHCUs) receive the best possible care, treatment and rehabilitation services (Mkhize & Kometsi 2008). The National Mental Health Policy Framework and Strategic Plan 2023–2030 (NMHPFSP) was reviewed and released in April 2023; the policy builds on the Constitution of South Africa and the MHCA. The policy aims to address all mental health conditions across the lifespan (Republic of South Africa 2023). The health budget allocated to mental health services is a very minimal budget of 5%. Budget affects human resources and infrastructure. Hlongwa and Sibiya (2019) highlight the shortage of psychiatric nurses, specialist psychiatrists and advanced psychiatric nurses as a hindering factor in integrating mental healthcare in PHCs in KwaZulu-Natal (KZN). There is a definitive need for ongoing training and support for PHC workers, supervision by specialists and emotional support to avoid burnout (Mkhize & Kometsi 2008).

The *Health Professions Act 56 of 1974* defines the scope of the physiotherapy profession and states that physiotherapists provide ‘treatment of physical ailments of psychiatric patients’ (Republic of South Africa 2009). The scope of the physiotherapy profession mentions physiotherapists managing PLWMI. Yet, the NMHPFSP, MHCA and Health Professions Council of South Africa (HPCSA) do not reference physiotherapists as part of the mental healthcare professional team in South Africa. The HPCSA has minimum standards for training for physiotherapy that outline the different areas of training along with the credits needed to complete the undergraduate training (Health Professions Council of South Africa 2023). Sixty-nine per cent of the countries in sub-Saharan countries have a mental health policy or plan (Vancampfort et al. 2018); only Namibia states that physiotherapy is part of the mental healthcare team and the profession has a role in mental health (Vancampfort et al. 2018). Our study aimed to explore the mental health content in the undergraduate physiotherapy programme at eight universities in South Africa and the perceptions of academic staff and stakeholders at an identified South African university about the relevance of mental health in the undergraduate physiotherapy programme.

## Research methods and design

Study design: Our study employed a concurrent-mixed method design, which consisted of: (1) the administration of a survey to academic leaders or lecturers undertaking mental health teaching at the university and (2) an online interview that included stakeholders and a focus group to gauge perceptions of academic staff at the identified university.

Study setting: Our study was conducted with eight South African universities offering the physiotherapy undergraduate programme. The universities that offer physiotherapy undergraduate programmes are the University of KwaZulu-Natal (UKZN), University of Pretoria, University of the Witwatersrand, University of the Free-State, Sefako-Makgatho University, University of Cape Town, University of Western Cape and Stellenbosch University. The study was conducted from January 2023 to November 2023.

### Phase 1: Administration of the survey

The course content survey was previously used to note the mental health content at universities in Australia and New Zealand (Connaughton & Gibson 2016b). The course content survey has seven questions that require a yes or no response. The survey looks at the basic overview of mental illnesses, the prevalence of mental illness, the pathophysiology, pharmacological management and communication strategies of students. The survey did not ask participants for any demographical information. Our study had a minimum of 2 participants based on a two-sided confidence level (1 aplha) of 95% and 80% power. An expected frequency of 50% was used for calculation because there is no data to rely on from South African literature. Ten per cent of the calculated minimum sample size was added to address incomplete responses.

### Phase 2: Focus group and interviews

Interviews were conducted with academic leaders and/or lecturers involved in mental health teaching, lecturers at the identified university and the HPCSA representatives. Interviews were conducted with six of the universities and one focus group with the lecturers at the identified university. The focus group consisted of three lecturers. The interviews were conducted online. The interviews and focus group consisted of four questions for the academic leader and/or lecturers involved in mental health training and lecturers at the identified university:

- Do you think the university undergraduate programme prepares the students to manage a PLWMI efficiently and effectively?
- Do students in the undergraduate physiotherapy programme have clinical exposure to managing a PLWMI?
- Have they been taught the assessment techniques and treatment modalities for PLWMI?
- Do you think it is crucial for the curriculum to include mental health training?

Two representatives on the Professional Board for Physiotherapy, Podiatry and Biokinetics of the HPSCSA participated in the interviews. The HPCSA representatives were asked the following questions:

- Regarding the statement in the HPCSA guidelines that ‘physiotherapists treat physical ailments of psychiatric patients’ ‘can you expand on what that entails?’
- Do you believe that physiotherapists should be treating patients with psychiatric diagnoses without a physical ailment?
- Do you believe that the HPCSA guidelines should be reviewed with consideration to include treatment of psychiatric patients without physical ailments, as there are physiotherapeutic interventions that physiotherapists can use?
- What depth of training about mental health do you think universities should be providing physiotherapy students to manage a patient effectively and efficiently with a mental illness?
- One of the important aspects of the physiotherapy profession is to promote a healthy lifestyle. How would a physiotherapist provide such information to a patient living with a mental illness if they are not familiar with mental illness?

### Data collection

Eight universities were recruited to participate in our study. Total population sampling was used to represent all the universities offering physiotherapy undergraduate training. Five of the universities and HPCSA requested additional ethical approval. The remaining universities accepted the ethical approval from UKZN. Once the ethical approval was granted, the academic leaders were contacted via email explaining the purpose of our study. The academic leaders either participated or nominated a lecturer from the department involved in mental health teaching. The online survey using Google Documents was sent via email. Informed consent was obtained when the participants completed the survey. When the participant completed the survey, an interview was arranged via Zoom. The interviews and focus group were approximately 30 min. The audio from the interviews was recorded. The interview with the academic leaders and focus group with the lecturers at UKZN aimed to gather more insight into the curriculum. The interview specifically aimed to determine whether students were adequately prepared to manage PLWMI after graduation and to assess whether the curriculum provided both theoretical and practical clinical experience in learning assessment and treatment skills. Additionally, the interview and focus group evaluated the relevance and importance of mental health training. The representatives of the HPCSA were interviewed to gain insight into the two documents that guide curriculum at universities: the scope of the physiotherapy profession (Republic of South Africa 2009) and the minimum standards for training physiotherapists (Health Professions Council of South Africa 2023).

### Data analysis

The quantitative analysis consisted of capturing the completed course content surveys on an MS Excel spreadsheet and representing the analysis graphically. The qualitative data recorded from the interviews and focus group were transcribed, coded and analysed using content analysis (Cote et al. 1993). NVivo version 14 software was utilised to enhance the data analysis process. NVivo’s robust tools for coding allowed for the creation and management of nodes (themes, ideas or concepts) and easy coding of the data from the interviews and focus group. The software’s support for automatic coding based on keywords saved time and increased consistency. Common themes and concepts were identified and grouped. Independent data coding, triangulation and peer debriefing were employed to ensure trustworthiness (Sparks 1998). NVivo’s querying tools facilitated the exploration of patterns and relationships within the data, while its data visualisation features helped in understanding and presenting findings more effectively. These checks, along with the use of NVivo, were essential in ensuring that the participants’ perspectives were accurately interpreted.

### Ethical considerations

Our study considered all ethical, legal and regulatory norms and standards of country, province and department in carrying out this research. This was performed by applying to the appropriate authorities: the Humanities and Social Sciences Research Ethics Committee, the Registrar at UKZN and the academic leader of the physiotherapy discipline at UKZN. Ethical approval was granted by the UKZN Humanities and Social Sciences Research Ethics Committee (reference no.: HSSREC/00004701/2022). Additional ethical approval was granted at the University of the Free State (UFS-HSD2023/0272/2507), University of Western Cape (UWCRP275299), Stellenbosch University institutional permission (IG-4020), University of Cape Town (HREC 123/2023) and Health Professions Council of South Africa (Research, 2023). Sefako-Makgatho University and the University of the Witwatersrand accepted UKZN ethical clearance.

Every participant was given detailed information about our study and informed that their participation was voluntary and that they could withdraw from our study at any time. Confidentiality, anonymity and privacy were maintained throughout our study. The research was carried out according to the principles of the Helsinki Declaration.

## Results

**FIGURE 1:**
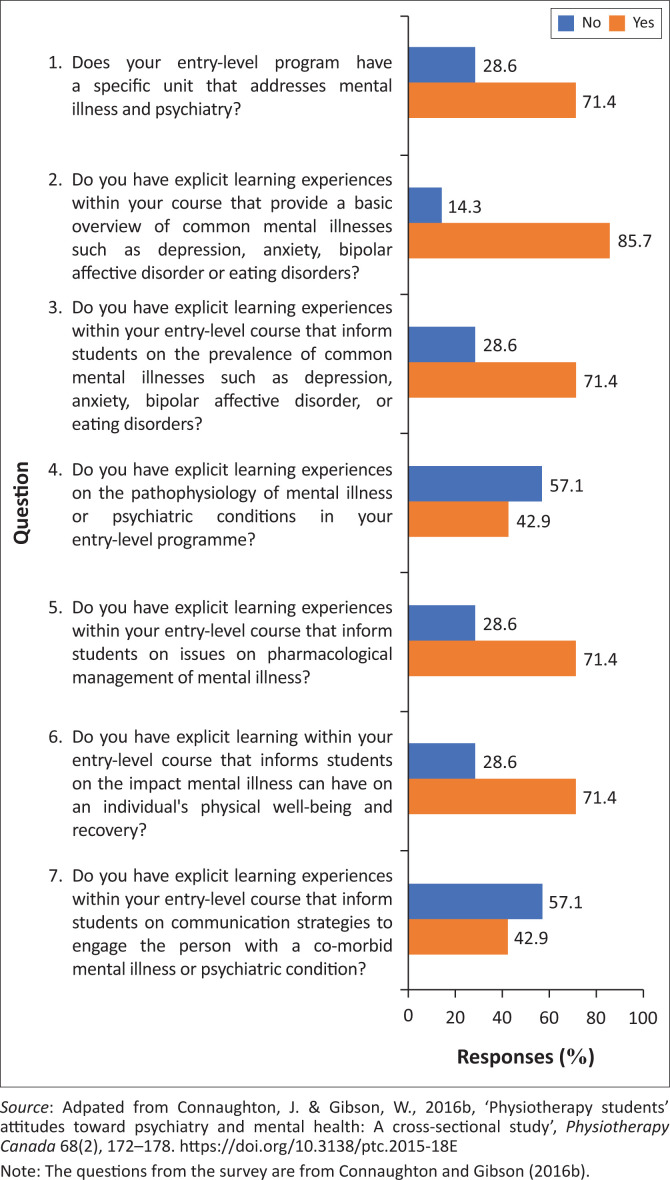
Course content survey.

Seven of the eight universities participated in completing the survey and interview. This represented 87.5% of the total population. Figure 1 depicts the results of the survey. The responses from questions 1, 2, 3, 5 and 6 show that the majority (71%–85.7%) of the universities were in positive consensus. The majority of the participants responded positively that there are programmes that specifically address mental illness and psychiatry, a basic overview of common mental illnesses, the prevalence of mental illness, pharmacological management and the impact of mental illness on physical health. Participants responded that they did not have explicit learning experiences on the pathophysiology of mental illness or psychiatric conditions and a course that informed students on communication strategies in the entry-level programme (57.1%). Three of the universities acknowledged not having learning experiences on the pathophysiology of mental illness and communication strategies. One of the three universities only had gaps in mental health training with regard to pathophysiology and communication strategies and was implementing the other areas of mental health training. Two of the three universities reported negative responses to the course content survey ranging from 71.4% to 85.7%, indicating that they did not provide adequate training for students.

The responses from academic leaders and/or lecturers who teach mental health, as well as the lecturers at UKZN, are depicted in Table 1. The interviews showed that one university had a theory and practical exposure for students to mental health, whereas four universities had neither theory nor practical exposure. Two of the universities educate students on assessment and treatment techniques. All seven of the universities agreed on the importance of having mental health content in the curriculum.

**TABLE 1:** Responses from academic leaders and/or lecturers teaching mental health and lecturers at an identified South African University.

Question	Thematic analysis	University no.	Illustrative quotes
Do you think the university’s undergraduate programme prepares the students to manage PLWMI efficiently and effectively?	Three universities reported that the students were not prepared. Two of the seven universities reported that they were prepared to varying extents. Two universities reported that students were prepared partially. All the universities have a psychology module in the first year of study.	University 1	‘There are no physiotherapy specific theory that is being taught.’
		University 3	‘The answer is very straightforward, no and I don’t think that they are prepared to, not in terms of the undergraduate curriculum to treat patients with mental disorders.’
Do students in the undergraduate programme have clinical experience in managing PLWMI?	Four universities reported that they do not have a clinical placement or theory component dedicated to mental health. Two of the universities reported that they had lectures on mental health: The first one had an hour-and-a-half lecture in 4th year about the role of physiotherapy in mental health. The second university has a lecture in the second year that is 6 h about mental health and pain management in 3rd and 4th year. One university had a theory and clinical exposure for students, but not all students participated in the clinical block, which is 5 weeks at a psychiatric hospital, and they had 6 h of examinable theory lectures.	University 1	‘If you ask me, they learn much more technically than in a lecture because it is hands on. And the feedback I get from the students is that the practical site is where they have learnt the most about the multi-disciplinary team.’
Have the students been taught assessment techniques and treatment modalities for PLWMI?	Five universities reported that they do not teach assessment or treatment modalities related to mental health, but they do focus on general communication strategies. Two universities reported that they teach students assessment and treatment modalities. A variety of content about mental health that includes communication strategies, health promotion, the role of physiotherapy in mental health, stigma and perceptions, psychosocial rehabilitation, assessment techniques and conducting classes, stress management, cognitive behavioural therapy and motivational interviewing.	University 4	‘Another golden thread that we pull through and we actually have in every year, we spend time in specific lectures dealing with communication.’
		University 6	‘I go through the types of treatment available, CBT, stress management, cognitive restructuring.’
		University 1	‘Communication is done across the clinical blocks, we coach students on effective interviewing skills, empathy, listening with empathy.’
Do you think having mental health content in the curriculum is crucial?	All the universities agreed on the importance of having mental health content in the curriculum, especially with the prevalence of mental illness in South Africa. One university recommended more practical sessions in the curriculum and more can be done to develop the mental health curriculum in physiotherapy.	University 1	‘Physiotherapists can assist patients besides using exercise. We do relaxation but not enough of it, deep breathing, yoga, Pilates but we need to change the constitution as it doesn’t identify physiotherapists as mental health care practitioners.’
		University 2	‘We need to give students the same techniques and the same skills in South Africa so we can evaluate the impact in our practice. We need a workforce that is multi-skilled; that’s the way forward.’
		University 3	‘The whole role of physiotherapy needs to be more recognised.’

PLWMI, people living with mental illness; no., number.

Table 2 depicts the responses from the interviews with the HPCSA representatives. The interview explored the *Health Professional Act 1974*, which is the scope of the profession for physiotherapy and the minimum standards for training for the undergraduate physiotherapy curriculum. The scope of the profession of physiotherapy can be interpreted as patients with mental illness treated by physiotherapists, but their focus is on treating the physical aspect. The scope of the profession of physiotherapy is currently under review and the treatment goal should be to improve the quality of life for every patient. The minimum standards for training do not compel training areas to universities but rather provide guidance for the curriculum.

**TABLE 2:** Responses from the Health Professions Council of South Africa representatives.

Question	Thematic analysis	HPSCA representative no.	Illustrative quotes
With regard to the statement in the HPCSA scope of the profession for physiotherapy (Republic of South Africa, 2009) that ‘physiotherapists treat physical ailments of psychiatric patients’.	The interpretation of the statement is that we treat patients with mental illness, but the focus is on the physical aspect.	HPCSA representative 2	‘We treat the physical ailments, physical impairments not the psychiatric illness so they could have a muscle spasm, muscle weakness or joint stiffness so we could treat those.’
Do you believe that physiotherapists should be treating patients with psychiatric diagnoses without a physical ailment?	The consensus was that the treatment goal for a patient is not based on the diagnosis but rather on improving the quality of life for the patient.	HPCSA representative 1	‘If you embark on exercise or physical interventions for the betterment of the patient’s mental health because we know that exercise and other potential manual techniques can have an influence on the brain as well as your mental health, then yes, it does fall within the scope.’
Do you believe that the HPCSA guidelines should be reviewed with consideration to include treatment of psychiatric patients without physical ailments, as there are physiotherapeutic interventions that physiotherapists can use?	The scope of the profession is currently under review. Physiotherapists can be involved in health promotion for primary or secondary complications.	HPCSA representative 1	‘We have a role of prevention even within our scope whether primary prevention.’
		HPCSA representative 2	‘The new scope will not speak to it specifically because it’s so broad, it includes anybody whose physical function and quality of life is affected by any disorder in their body.’
What depth of training about mental health do you think universities should be providing physiotherapy students to effectively and efficiently manage a patient with a mental illness?	The HPCSA does not dictate the content of the curriculum to the universities. It is merely a guideline for the curriculum. The institutions have academic freedom.	HPCSA representative 2	‘The whole approach in education is to get the students to think and what our techniques can do with the physiological effects and then link that to what the patient needs.’
One of the important aspects of the physiotherapy profession is to promote a healthy lifestyle. How would a physiotherapist provide such information to a patient living with a mental illness if they are not familiar with mental illness?	There are a lot of conditions that the students may not get exposure to. The universities should provide the basic pathophysiology of conditions.	HPCSA representative 2	‘There is no way you can cover all those conditions. So, what’s happening in education is that we are moving away from this tick box of having to cover all these conditions to more of understanding the process of engaging with the patient, building therapeutic relationships, decision making, clinical reasoning around interventions, prescriptions, regardless of whether you heard about the condition.’

HPCSA, Health Professions Council of South Africa; no., number.

## Discussion

Our study investigated the mental health content in the undergraduate physiotherapy programme in South Africa. Seven of the eight universities offering the physiotherapy undergraduate programme in South Africa participated in the survey, interviews and focus group. All the universities agreed to have a psychology module, but this was not profession-specific. The data revealed that the mental health content in the curriculum was crucial for physiotherapy students, but the nature and provision were diverse and inconsistent across the South African universities.

The course content survey revealed two main areas of training that were limited in most universities: the pathophysiology of mental illness and communication strategies. The limited knowledge of the pathophysiology of mental illness was also found in studies conducted in Australia and New Zealand (Connaughton & Gibson 2016a, 2016b) and KZN, South Africa (Hooblaul, Cobbing & Daniels 2020). The importance of being educated about the pathophysiology of mental illness will assist physiotherapy students and physiotherapists in interacting using a person-centred approach and bridge the gap between physical and mental health (Connaughton & Gibson 2016a).

The limited knowledge of communication strategies to engage with PLWMI was found in studies in Paraguay (Almirón et al. 2020), Australia and New Zealand (Connaughton & Gibson, 2016a, 2016b) and KZN in South Africa (Hooblaul et al. 2020). A study in the United States of America investigated the impact of severe mental illness (SMI) service learning (a course that provided interaction with patients with SMI using the person-centred approach) and the participants reported a great improvement in their communication skills with patients (Karyczak et al. 2020). The participants reported an increase in patience, building a rapport with patients and confidence in managing patients with SMI (Karyczak et al. 2020). An essential reason for effective communication strategies is compliance with treatment programmes when patients’ psychological needs are supported (Murray et al. 2015). The participants in the focus group expressed that there was no specific module on communication but that it was integrated into the clinical training and not specifically for the management of PLWMI.

None of the universities had similar mental health content or training. One university that provided both theoretical and clinical education emphasised the importance of having students’ clinical exposure to patients with mental health problems. Implementing a curriculum with mental health in physiotherapy will avoid stigmatising attitudes and behaviours; even though they may not directly manage the mental illness, they will be able to manage a patient with a psychiatric co-morbidity or chronic illness (Yildirim et al. 2015). Two studies proved that having examinable theory and clinical training improved physiotherapy students’ attitudes towards mental health (Bhise et al. 2016; Probst & Peuskens 2010). Bhise et al. (2016) explored the difference in thepsychiatry curriculum of undergraduate medical and physiotherapy students on physiotherapy students’ attitudes towards psychiatry. Probst and Peuskens (2010) explain the attitudes of physiotherapy students before and after a 67-h course entitled ‘Pathology and psychomotor rehabilitation for patients with psychopathological illnesses’ were significantly improved. The course content was theoretical and practical. Our study concludes that student attitudes were more positive after the training. Course and practice could prevent mental health stigma in physiotherapy students (Probst & Peuskens 2010).

Every university strongly agrees that having mental health content in the physiotherapy undergraduate curriculum is vital. Reviewing a curriculum is a mammoth task as the curriculum is condensed. The impact and the need for mental health knowledge for physiotherapists and physiotherapy students on attitudes and perceptions have been observed in studies globally and in South Africa (Almirón et al. 2020; Andrew et al. 2019; Bhise et al. 2016; Connaughton & Gibson, 2016a, 2016b; Dandridge et al. 2014; Gunduza, Lord & Keller 2023; Hooblaul et al., 2020; Karyczak et al., 2020; Lennon et al. 2020; Overmeer et al. 2009; Prasanna, Kumari & Vigneshwar 2021; Probst & Peuskens 2010; Rutvi, Shah & Parikh 2022; Sarin 2003; Yildirim et al. 2015; Zechner et al. 2022). The physiotherapy curricula vary worldwide and only a few show interest in mental health, so students may not get the opportunity to develop skills in mental health (Rathi et al. 2023).

The members of the Board of Physiotherapy, Podiatry and Biokinetics provided insight into the two documents that guide the curriculum for the physiotherapy profession (Health Professions Council of South Africa 2023; Republic of South Africa 2009). The scope of the profession for physiotherapy states the treatment of psychiatric patients with physical ailments. The participants emphasised that the management of a patient should be focussed on improving the quality of life rather than a diagnosis. This further justifies the use of a biopsychosocial model. Each person’s rehabilitation needs, together with social, psychological and work-related needs, should be considered (Probst & Skjaerven 2017).

There should be consistency in the physiotherapy curriculum in South Africa so that every graduate physiotherapist has the same technical skills and knowledge. The high prevalence of mental illness in South Africa may lead to patients with co-morbidities or chronic conditions seeking physiotherapy interventions at some stage in their lives. Mental health specialists cannot solely meet the needs of the population and therefore healthcare professionals should have basic mental health and psychiatry knowledge (Bhise et al. 2016).

### Strengths and limitations

This is the first study to explore the mental health content in physiotherapy undergraduate programmes in South Africa. The response rate for our study was good as only one university did not participate in the study. The ethical approval requested from five of the universities was a lengthy process and resulted in the study spanning over a year. A limitation of our study was that it did not go into detailed information on the mental health content at the universities that offered it.

### Recommendations

The recognition of physiotherapy in mental health is pertinent to address the gap in the curricula by reviewing policies in South Africa and the acceptance of physiotherapists as mental healthcare practitioners. The prevalence of mental illness and the gap in education on this should propel universities to initiate mental health training for physiotherapy students.

## Conclusion

Our study’s findings showed the disparities in the mental health content at the universities in South Africa. The physiotherapy students should have basic knowledge and clinical exposure to mental health; this will improve attitudes and reduce stigmatising behaviours. The practice of a biopsychosocial approach can be a challenge without adequate knowledge about mental health. A multiskilled workforce will address the shortage of human resources experienced in the South African healthcare system. Literature from international studies has proven the importance of having mental health content for both the patient and the physiotherapist. The curriculum at the universities in South Africa needs to be reviewed to include mental health.
